# The effect of alcohol withdrawal therapy on gut microbiota in alcohol use disorder and its link to inflammation and craving

**DOI:** 10.1111/acer.70128

**Published:** 2025-08-23

**Authors:** Phileas J. Proskynitopoulos, Sabrina Woltemate, Mathias Rhein, Isabell Böke, Jannis Molks, Sebastian Schröder, Hans‐Udo Schneider, Stefan Bleich, Helge Frieling, Robert Geffers, Alexander Glahn, Marius Vital

**Affiliations:** ^1^ Department of Psychiatry, Social Psychiatry and Psychotherapy Hannover Medical School Hannover Germany; ^2^ Institute for Medical Microbiology and Hospital Epidemiology Hannover Medical School Hannover Germany; ^3^ Department of Psychiatry and Psychotherapy Ruhr‐University Bochum Campus‐OWL Lübbecke Lübbecke Germany; ^4^ Genome Analytics Helmholtz Centre for Infection Research Braunschweig Germany

**Keywords:** alcohol use disorder, alcohol withdrawal, gut‐microbiome, neuroinflammation

## Abstract

**Background:**

Alcohol use disorder (AUD) is linked to changes in the function and composition of the human gut microbiome (GM). The GM affects inflammation by producing anti‐inflammatory molecules such as short‐chain fatty acids (SCFA), in particular butyrate, which are linked to appetite regulation, a mechanism involved in alcohol craving. This study investigates changes in GM composition and functional capacity to produce SCFA during alcohol withdrawal and their link to inflammation and craving.

**Methods:**

Sixty‐three patients (mean age 48, SD = 12) with AUD were enrolled. We collected stool (*n* = 63) and blood (*n* = 48) during the first 48 h (timepoint A) of withdrawal therapy and between Days 10–14 (timepoint B). Microbiota were analyzed using shotgun metagenomics along with bacterial load determinations. TNF‐α, IL‐6, IL‐8, and IL‐10 were measured in plasma.

**Results:**

Bacterial diversity (species richness, Shannon Index) did not change significantly throughout withdrawal, while overall bacterial load increased. Abundances of several taxa changed, and the overall community composition during withdrawal was approaching those of healthy controls; the potential to synthesize butyrate, a key SCFA, increased. However, it remained at lower levels compared with controls. Both diversity parameters correlated with cell concentrations and the butyrate pathway at baseline. The latter was negatively associated with IL‐6 at baseline. IL‐8 and IL‐10 levels decreased significantly during withdrawal, as did craving, which was linked to abundance alterations of six species and IL‐8.

**Conclusions:**

Alcohol withdrawal affected GM composition and increased concentration of the butyrate pathway along with overall bacterial load. Changes in bacterial composition and the butyrate production capacity demonstrate a shift toward healthier microbiota during withdrawal therapy. Changes in some species and IL‐8 were linked to alcohol craving, replicating findings of previous studies. Our study adds new findings helping to understand the microbiome–gut–brain axis.

## Graphical abstract

This study investigates the effect of alcohol withdrawal therapy on the gut microbiome and its relationship to specific interleukins and alcohol craving. Alcohol withdrawal affected microbiome composition and increased the concentration of the butyrate pathway, along with the overall bacterial load. Changes in bacterial composition and the butyrate production capacity demonstrate a shift toward healthier microbiota. Changes in some species and IL‐8 were linked to alcohol craving. Our study adds new findings helping to understand the microbiome–gut–brain axis.

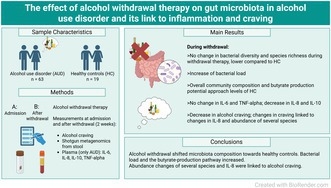

## INTRODUCTION

Alcohol use disorder (AUD) is one of the most significant global health burdens (World Health Organization, 2018). Although substantial steps were taken toward a general neurobiological characterization of AUD and substance use disorders in general (Koob & Volkow, 2016), new pharmacological therapies and therapeutic targets are scarce.

In the last decade, an increasing number of studies have published evidence on the use of probiotic supplements/drugs for the treatment of psychiatric diseases (Hillemacher et al., 2018). Because of rigorous research, it is now well‐known that changes in the human microbiome are linked to AUD (Leclercq, 2024) and alcohol‐associated liver disease (ALD) (Hsu & Schnabl, 2023).

The liver, being the primary organ draining the blood from the gut and metabolizing alcohol, is a key player when it comes to understanding the relationship between microbiome changes, inflammation, and impaired brain function seen in AUD and ALD (Bajaj, 2019). Alcohol is well‐known to cause gut dysbiosis, endotoxemia, and inflammation that increases the risk for the development of ALD (Bajaj, 2019; Hartmann et al., 2015; Jew & Hsu, 2023; Singhal et al., 2021). Specific changes in human gut microbiota are linked to an increased risk of developing ALD, liver cirrhosis, and hepatocellular carcinoma (Hsu & Schnabl, 2023). A recent review has thoroughly examined the role of interorgan cross talk between the gut and the liver that is mediated by interleukins that play a central role in the development of alcoholic steatohepatitis, a disease significantly diminishing prognosis in AUD patients (Gao et al., 2019). To this end, one study using shotgun metagenomics has identified different bacterial taxa linked to AUD and liver cirrhosis. The authors conclude that shifts in the microbial community structure suggest a strong negative influence of both AUD and associated liver function on gut microbiota (Dubinkina et al., 2017).

Several studies have described a relationship between changes in gut microbiota and human behavior. The microbiome affects neuroinflammation associated with alcohol consumption and interferes with central neurochemistry and behavior (Leclercq et al., 2019). For example, microbiome alterations have been related to impaired sociability in AUD patients (Leclercq et al., 2020) and binge drinking in healthy young adults (Carbia et al., 2023). In one study during withdrawal therapy, the authors describe that changes in intestinal permeability were associated with depression, anxiety, and craving (Leclercq, Matamoros, et al., 2014).

Specific bacterial taxa have been reported to be altered in patients suffering from AUD. In essence, *Bifidobacteria* and *Lactobacilli* are two bacterial families that show promising effects on the dysbiosis caused by alcohol consumption, as they are often found to be significantly lower in AUD patients when compared to controls (Leclercq et al., 2017). In one study, *Bifidobacterium bifidum* and *Lactobacillus plantarum* were administered once‐daily for five consecutive days, and the authors found that the administration of those bacteria led to a more significant reduction in liver enzymes in those patients suffering from an alcohol‐induced liver injury (Kirpich et al., 2008). In different in vitro and in vivo studies in both animals and humans, *Bifidobacteria* and/or *Lactobacilli* reduced the secretion of several interleukins such as TNF‐α, IL‐8, or IL‐10 production (Bai et al., 2004; Choi et al., 2008; Imaoka et al., 2008; Messaoudi et al., 2011; Ren et al., 2013; Zhang et al., 2005). According to Leclercq, De Saeger, et al. (2014), IL‐8 positively correlated with alcohol drinking amount and craving in 63 patients during alcohol detoxification. Possible therapeutic usage of those two taxa as probiotics in alcohol‐dependent patients is also discussed by Leclercq et al. (2017). Based on those findings, the hypothesis arises that bacteria affecting entero‐inflammation and interleukin secretion also have an influence on craving. However, the exact underlying mechanisms are still to be revealed.

A central gut microbiota (GM)‐derived molecule for human health is butyrate. Butyrate, a short‐chain fatty acid (SCFA), provides energy to the epithelium, acts anti‐inflammatory, and affects diverse metabolic routes throughout the body, including the gut–liver as well as the gut–brain axis (Amiri et al., 2022; Karim et al., 2024; Li et al., 2018; Nicholson et al., 2012; Smith et al., 2013; Vital et al., 2017). Previous research has shown that the abundance of butyrate‐producing bacteria directly leads to an increase in butyrate production (Kircher et al., 2022). In the context of AUD, it was shown to ameliorate chronic central nervous damage caused by alcohol through suppressing microglia‐mediated neuroinflammation (Wei et al., 2023). In one animal study, feeding sodium butyrate did not alter basal ethanol intake levels but lowered ethanol preference in a two‐bottle choice (Reyes et al., 2022). Furthermore, a few studies have investigated the relationship between butyrate synthesis and alcohol craving, for example, in young binge drinkers (Carbia et al., 2023), suggesting a direct link via appetite‐controlling hormones, such as leptin (Bach et al., 2021) and ghrelin, that are known to be partly regulated by butyrate (Koopmann et al., 2018).

As of today, most studies investigating gut microbiota in AUD used 16S rRNA gene sequencing to assess bacterial diversity and composition. However, to gain insights at high taxonomic resolution as well as on functions, it is important to apply shotgun metagenomics (Huttenhower et al., 2012; Luo et al., 2011). In this study, we performed metagenomics analyses that were complemented by flow cytometric measurements on bacterial concentrations in order to obtain quantitative insights at high resolution. With this in hand, we tested how gut microbiota composition and the butyrate production capacity changed during withdrawal treatment and how it associates with interleukin levels as well as behavioral parameters such as craving and alcohol intake.

## MATERIALS AND METHODS

### Participants

This study is part of a large prospective research project on the association between the human microbiome and AUD during withdrawal therapy. The study was approved by the Ethics Committee at Hannover Medical School (Nr. 7909_BO_S_2018) and followed the declaration of Helsinki. All patients were recruited at the Department of Psychiatry, Social Psychiatry, and Psychotherapy of Hannover Medical School and the Department of Psychiatry and Psychotherapy at Minden/Lübbecke. Before enrollment, each participant gave written informed consent to participate in the study. In the patient group, all participants suffered from alcohol dependence according to DSM‐IV and ICD‐10 and planned to undergo a qualified withdrawal therapy to reach abstinence from alcohol. Qualified withdrawal therapy in Germany normally lasts 14–21 days. Withdrawal symptoms are usually treated with benzodiazepines (mainly oxazepam, lorazepam or diazepam) or clomethiazole (Kiefer et al., 2021). The patients were not obliged to participate in the study to undergo qualified withdrawal therapy. We excluded patients with anorexia, diabetes mellitus type I, Crohn's disease, or ulcerative colitis, severe liver disease such as liver cirrhosis or acute alcoholic steatohepatitis, and any comorbid psychiatric disorders that could prohibit informed consent. Furthermore, we excluded patients who recently received antibiotic treatment (defined as during the first 2 weeks before enrollment). After enrollment, all patients underwent a detailed physical examination by a professional observer, routine laboratory testing, and urine drug screening. Blood and stool samples were taken during the first 48 h of withdrawal therapy (timepoint A) and after the tenth day of withdrawal therapy (days 10–14, timepoint B). Then, all of the blood samples were centrifuged and stored at −80°C immediately after collection. Stool samples were directly stored at −20°C.

Psychometric assessment at admission included general demographics and specific measurements concerning alcohol use. In detail, the AUDIT (Saunders et al., 1993) was used for the assessment of AUD. The extent of alcohol craving was obtained using the Obsessive Compulsive Drinking Scale (Anton et al., 1995) (OCDS) and Penn Alcohol Craving Scale (Flannery et al., 1999) (PACS), which provide a well‐validated and reliable assessment.

We additionally assessed the impact of withdrawal by comparing results derived from patients with AUD with metagenomics data from healthy controls (*n* = 19) from a previous study (Buttler et al., 2025; Kircher et al., 2022). The mentioned study was approved by the local ethics committee (#8566_BO_K_2019).

### Microbiological analysis

Samples were subjected to metagenomic (“shotgun sequencing”) analyses, where extracted DNA was treated with the Nextera DNA Flex Library Prep Kit (Illumina) and sequenced on Illumina NovaSeq6000 (sequencing was done at Genome Analytics, Helmholtz Centre for Infection Research). Raw reads were quality‐filtered using Kneaddata (Huttenhower lab; v0.7.2) and subjected to MetaPhlAn4 yielding taxonomic compositions. For determining pathways of specific key functions, namely, for the production of the SCFA butyrate and propionate, custom gene catalogs based on UHGG.v2 representative genomes were used for mapping via BBmap (from JGI; v38.22; paired‐end mode) as described previously (Kircher et al., 2022). All bioinformatics analyses were performed on MHH's High‐Performance Computer Sequencing Cluster (HPCSeq).

Total bacterial concentrations (bacteria per gram stool) were analyzed by flow cytometry as described previously (Kircher et al., 2022). In brief, 200 mg of stool was diluted 5 < 0.001× and filtered (preseparation filter (30 μM) from Miltenyi Biotec). Solutions were subsequently stained with SYBR Green I (ThermoFischer) and measured on a MACSQuant Flow Cytometer.

### Statistical analysis

For all statistical analysis, we used R (v4.2.2) and the Statistical Package for the Social Sciences Version 29 (SPPS, IBM, Armonk, NY, USA).

To compare the richness of microbial communities, we compared the Shannon index using a t‐test for paired samples with a *p*‐value (two‐tailed) below the alpha level of 5% being considered statistically significant. We used the nonparametric Wilcoxon's test for paired samples to compare bacterial load, butyrate, propionate, and the different cytokines and craving between timepoint A and B. Statistical analyses of microbial taxa during withdrawal were performed in R (v4.2.2) applying linear mixed‐effect models (lmer from the lme4 (v1.1–34) package) on compositional data using log transformations (log10(data+1)) including patient as a random effect. Results were corrected for multiple testing using *fdrtools* (v1.2.17), where a *q*‐value <0.05 was considered significant. For correlation analyses between species, cytokines, and clinical parameters, we used Spearman's correlation coefficient, filtering results by significance (*p* < 0.01) and correlation coefficient (±0.3).

## RESULTS

### Patient characteristics

A total of 63 patients were recruited in both centers across Germany. The sample consisted of 44 males and 19 females. For an overview of patient characteristics, see Table 1.

**TABLE 1:** Demographics.

Sex (m vs. f)	44 vs. 19
Smoking (yes vs. no)	52 vs. 9
Age (*n*, mean, SD)	63, 47.94, 12.24
BMI (*n*, mean, SD)	54, 27.21, 5.38
Daily intake in g (*n*, mean, SD)	61, 189.52, 123.26
Number of withdrawals (*n*, mean, SD)	59, 5.44, 7.54
Breath alcohol concentration at baseline per mille (*n*, mean, SD)	23, 0.95, 1.13
Interleukin 6 in pg/mL Timepoint A (*n*, mean, SD)	48, 10.75, 32.91
Interleukin 6 in pg/mL Timepoint B (*n*, mean, SD)	48, 6.96, 13.47
Interleukin 8 in pg/mL Timepoint A (*n*, mean, SD)	48, 99.96, 118.39
Interleukin 8 in pg/mL Timepoint B (*n*, mean, SD)	48, 70.19, 86.20
Interleukin 10 in pg/mL Timepoint A (*n*, mean, SD)	48, 35.92, 122.01
Interleukin 10 in pg/mL Timepoint B (*n*, mean, SD)	48, 13.23, 19.61
TNF‐α in pg/mL Timepoint A (*n*, mean, SD)	48, 132.29, 139.30
TNF‐α in pg/mL Timepoint B (*n*, mean, SD)	48, 136.46, 174.53
AUDIT (*n*, mean, SD)	56, 29.04, 7.19
PACS Timepoint A (*n*, mean, SD)	48, 18.42, 7.08
PACS Timepoint B (*n*, mean, SD)	57, 8.35, 6.99
OCDS Timepoint A (*n*, mean, SD)	41, 23.17, 6.56
OCDS Timepoint B (*n*, mean, SD)	49, 13.71, 7.39

Abbreviations: AUDIT, alcohol use disorder identification test; BMI, body mass index; g, grams; f, female sex; m, male sex; *n*¸number of samples; OCDS, obsessive compulsive drinking scale; PACS, penn alcohol craving scale; SD, standard deviation; TNF, tumor necrosis factor.

### Microbiota composition

**FIGURE 1:**
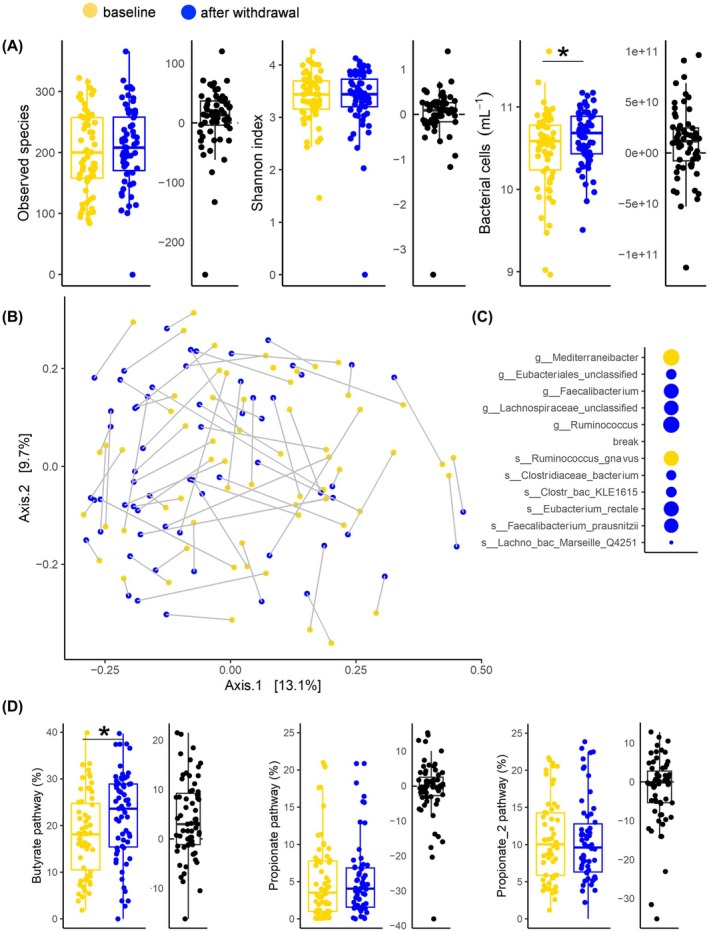
Microbiota changes during withdrawal therapy. Panel (A) gives diversity and bacterial load (bacteria per gram stool) data at baseline (yellow) and after withdrawal (blue) as box plots. In (B) metric multidimensional scaling analysis of communities (on species level) is depicted with samples from each patient connected, whereas panel (C) indicate changes of specific key taxa (blue: Increased after withdrawal; yellow increased at baseline; dot sizes relate to effect sizes of linear regression analyses with higher effect sizes translating into larger dot sizes). Panel (D) depicts abundances of the butyrate‐ and two propionate‐producing pathways as percentages of genomes exhibiting respective pathways as box plots. Plots in (A) and (D) containing black dots show mean difference between the two time points, while yellow dots depict timepoint A and blue timepoint B. Significant differences are indicated by asterisks. *p < 0.05.

Bacterial diversity, determined as species richness and the Shannon index, did not change significantly throughout withdrawal, whereas a significant increase in bacterial load was observed (*p* = 0.021) (Figure 1A). The bacterial composition remained subject‐specific throughout the experiment (Figure 1B). However, several taxa changed their relative abundances. On the species level, Ruminococcus *gnavus* decreased significantly, while abundances of *Clostridiales bacterium KLE1615*, *Eubacterium rectale*, *Clostridiaceae bacterium, Faecalibacterium prausnitzii*, and *Lachnospiraceae bacterium Marseille Q4251* increased (Figure 1C). On the genus level, the abundance of *Mediterraneibacter* (to which *Ruminoccocus gnavus* belongs) decreased significantly during withdrawal, while *Eubacteriales unclassified*, *Lachnospiraceae unclassified*, *Faecalibacterium*, and *Ruminococcus* increased (Table 2).

**TABLE 2:** Changes in bacterial abundance.

Bacteria	Estimate	*p*	*q*
f_Eubacteriales_unclassified	0.295	<0.001	0.009
f_Oscillospiraceae	0.425	<0.001	0.018
f_Erysipelotrichaceae	−0.257	0.001	0.042
g_Mediterraneibacter	−0.508	<0.001	0.003
g_Eubacteriales_unclassified	0.163	<0.001	0.021
g_Lachnospiraceae_unclassified	0.375	<0.001	0.023
g_Ruminococcus	0.494	<0.001	0.023
g_Faecalibacterium	0.401	0.001	0.026
s_Ruminococcus_gnavus	−0.416	<0.001	0.010
s_Clostridiales_bacterium_KLE1615	0.176	<0.001	0.010
s_Eubacterium_rectale	0.416	<0.001	0.028
s_Clostridiaceae_bacterium	0.153	<0.001	0.040
s_Lachnospiraceae_bacterium_Marseille_Q4251	0.034	<0.001	0.044
s_Faecalibacterium_prausnitzii	0.366	0.001	0.060

*Note*: Significant changes in abundances from time point A to time point B. The calculated estimates are based on logarithmic values.

Abbreviations: f, family; g, genus; s, species.

Diversity was significantly higher in healthy controls than in patient samples based on both species richness and Shannon index (*p* < 0.01) along with a significantly different composition based on PERMANOVA analysis (*p* < 0.01) (Figure S1). Importantly, the Bray–Curtis dissimilarity based on whole communities between controls and patients decreased significantly from timepoint A to B (*p* < 0.01) demonstrating that withdrawal promoted community alterations toward a healthy state (Figure S1).

### Specific microbiota function

We took a detailed look into abundances of SCFA‐producing pathways as those metabolites are hypothesized to play a key role in the microbiota–brain axis. The potential to synthesize butyrate did increase during withdrawal therapy, whereas pathways associated with propionate did not change in their abundances (*p* < 0.001 for butyrate and *p* = 0.951 and *p* = 0.370 for Propionate 1 and Propionate 2, respectively) (Figure 1D). Both diversity parameters did correlate with the butyrate pathway and cell concentrations (for Shannon *p* < 0.1) at timepoint A; bacterial load and the butyrate pathway correlated as well (data not shown). At the end of withdrawal, the number of bacteria producing butyrate (timepoint B, mean = 22.16%, standard deviation (SD) = 9.41%) remained lower compared with HC (mean = 29.86%, SD = 8.28%, *p* < 0.01) (Figure S1).

### Inflammatory parameters

IL‐8 and IL‐10 levels decreased significantly during the course of withdrawal (*p* = 0.015; *p* = 0.007), while there was no change in TNF‐α and IL‐6 levels (*p* = 0.965; *p* = 0.421, respectively).

**FIGURE 2:**
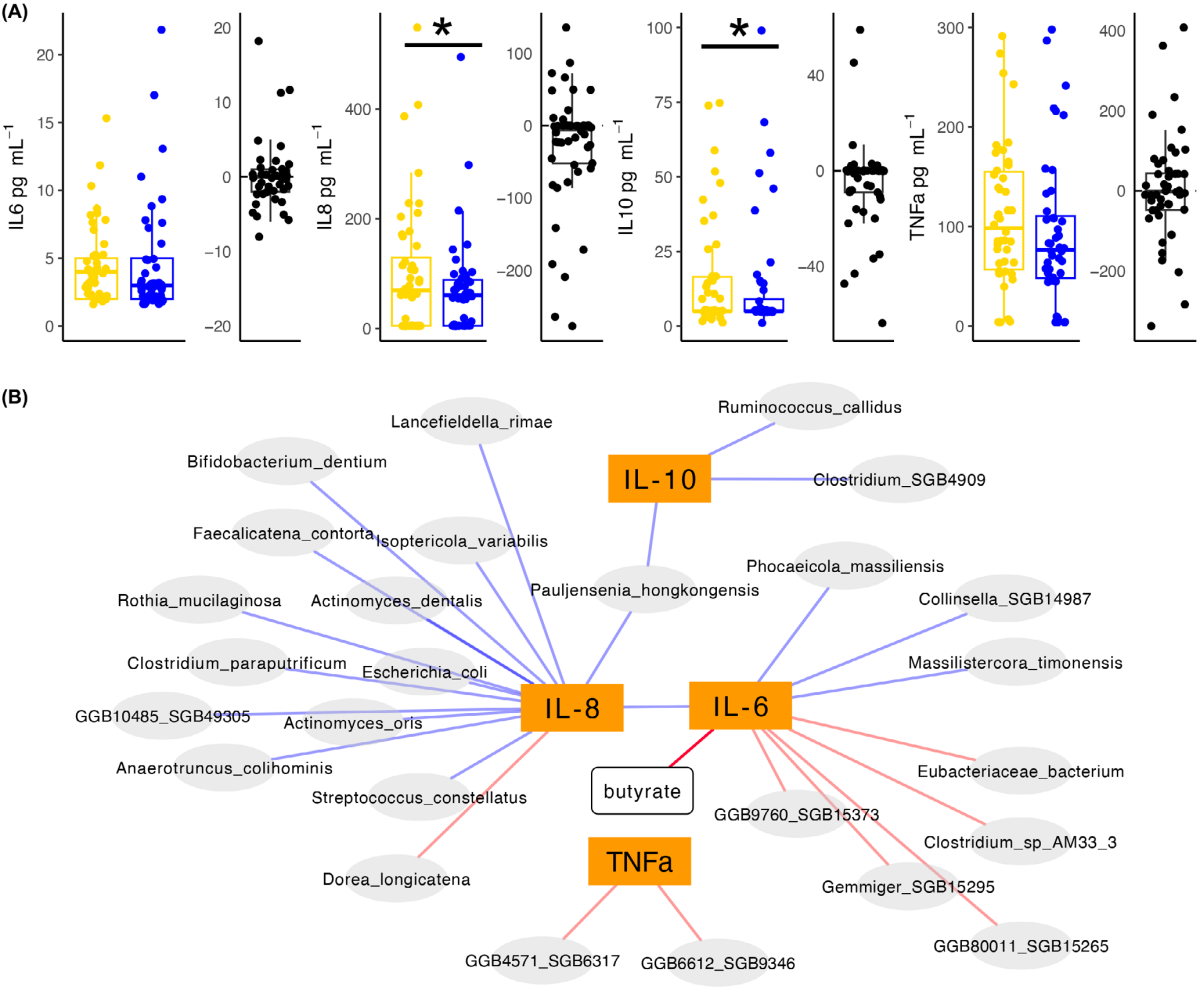
Changes in cytokine concentrations during withdrawal therapy. Panel (A) gives cytokine levels at baseline (yellow) and after withdrawal (blue) as box plots, where plots containing black dots show mean difference between the two time points. In (B) correlations of cytokine levels with abundances of different species as well as with the butyrate production potential at baseline are shown. Colors of edges refer to positive (blue) and negative (red) correlations. Significant differences are indicated by asterisks. *p < 0.05.

At baseline, IL‐6 and IL‐8 levels correlated with each other, whereas IL‐10 and TNF‐α were not associated with other markers. Abundances of several species were associated with cytokine concentrations, and the butyrate‐producing pathway correlated negatively with IL‐6 levels (Figure 2B; for details, see Table 3). Propionate pathways did not correlate with any cytokine concentrations (data not shown).

**TABLE 3:** Correlations between species and interleukins at baseline.

Row	Column	*ρ*	*p*
s_Dorea_longicatena	IL‐8	−0.382	0.007
s_Streptococcus_constellatus	IL‐8	0.430	0.002
s_Clostridium_paraputrificum	IL‐8	0.393	0.006
s_Actinomyces_oris	IL‐8	0.412	0.004
s_Rothia_mucilaginosa	IL‐8	0.422	0.003
s_Bifidobacterium_dentium	IL‐8	0.484	0.000
s_GGB10485_SGB49305	IL‐8	0.518	0.000
s_Pauljensenia_hongkongensis	IL‐8	0.403	0.005
s_Anaerotruncus_colihominis	IL‐8	0.418	0.003
s_Lancefieldella_rimae	IL‐8	0.373	0.009
s_Faecalicatena_contorta	IL‐8	0.397	0.005
s_Actinomyces_dentalis	IL‐8	0.435	0.002
s_Isoptericola_variabilis	IL‐8	0.402	0.005
s_Escherichia_coli	IL‐8	0.372	0.009
s_Phocaeicola_massiliensis	IL‐6	0.398	0.005
s_GGB80011_SGB15265	IL‐6	−0.415	0.003
s_Gemmiger_SGB15295	IL‐6	−0.403	0.005
s_Eubacteriaceae_bacterium	IL‐6	−0.387	0.007
s_GGB9760_SGB15373	IL‐6	−0.444	0.002
s_Massilistercora_timonensis	IL‐6	0.382	0.007
s_Clostridium_sp_AM33_3	IL‐6	−0.455	0.001
s_Collinsella_SGB14987	IL‐6	0.374	0.009
IL‐8	IL‐6	0.420	0.003
s_Pauljensenia_hongkongensis	IL‐10	0.443	0.002
s_Ruminococcus_callidus	IL‐10	0.448	0.001
s_Clostridium_SGB4909	IL‐10	0.403	0.005
s_GGB4571_SGB6317	TNF‐α	−0.454	0.001
s_GGB6612_SGB9346	TNF‐α	−0.384	0.007

Abbreviations: IL, interleukin; s, species; TNF, tumor necrosis factor.

### Clinical parameters

**FIGURE 3:**
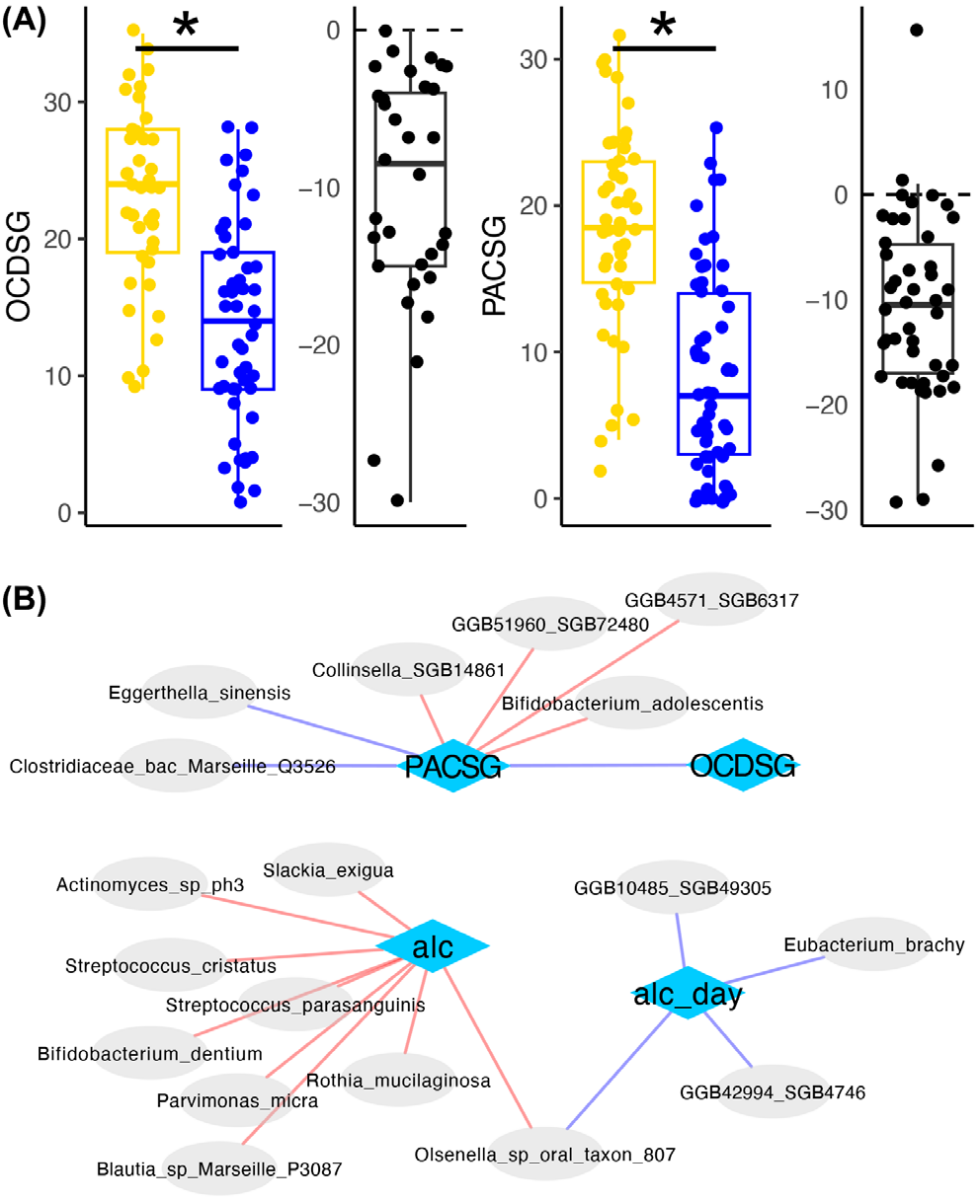
Changes in total craving scores, as assessed with the obsessive compulsive drinking scale (OCDSG) and penn alcohol craving scale (PACSG) during withdrawal therapy. Panel (A) gives scores at baseline (yellow) and after withdrawal (blue) as box plots, where plots containing black dots show mean difference between the two time points. In (B) their correlation with abundances of different species at baseline are shown; associations between species and breath alcohol concentration (alc) and daily intake (alc_day) are given as well. Colors of edges refer to positive (blue) and negative (red) correlations. Significant differences are indicated by asterisks. *p < 0.05.

Craving (measured with both the PACS and the OCDS) decreased significantly during withdrawal therapy (*p* < 0.001; *p* < 0.001, respectively) (Figure 3A).

At baseline, daily intake was associated with the abundance of *GGB10485 SGB49305, Eubacterium brachy, GGB42994 SGB4746, Olsenella sp oral taxon 807*, and alcohol concentration at admission was associated with the abundance of *Streptococcus parasanguinis, Rothia mucolaginosa, Slackie exigua, Bifidobacterium dentium, Actinomyces sp ph3, Parvimonas micra, Olsenella sp oral taxon 807, Streptococcus cistatus*, as well as *Blautia sp Marseille P3087*, respectively. (Figure 3B; Table 4). Also, PACS score was linked to abundances of the following species: *Collinsella SGB14861, Bifidobacterium adolescentis, Clostridiaceae bacterium Marseille Q 3526, Eggerthella sinensis, GGB4571 SGB6317*, *and GGB51960 SGB72480* (Figure 3B; Table 4). There was no correlation between SCFA pathways and clinical parameters at baseline.

**TABLE 4:** Correlation between species and psychometrics at baseline (timepoint A).

Row	Column	*ρ*	*p*
s_Collinsella_SGB14861	PACS	−0.390	0.006
s_Bifidobacterium_adolescentis	PACS	−0.379	0.008
s_Clostridiaceae_bacterium_Marseille_Q3526	PACS	0.398	0.005
s_Eggerthella_sinensis	PACS	0.379	0.008
s_GGB4571_SGB6317	PACS	−0.387	0.007
s_GGB51960_SGB72480	PACS	−0.398	0.005
OCDS	PACS	0.478	0.006
s_GGB10485_SGB49305	daily intake	−0.340	0.007
s_Eubacterium_brachy	daily intake	−0.364	0.004
s_GGB42994_SGB4746	daily intake	−0.336	0.008
s_Olsenella_sp_oral_taxon_807	daily intake	−0.330	0.009
s_Streptococcus_parasanguinis	BAC	0.546	0.007
s_Rothia_mucilaginosa	BAC	0.548	0.007
s_Slackia_exigua	BAC	0.551	0.006
s_Bifidobacterium_dentium	BAC	0.597	0.003
s_Actinomyces_sp_ph3	BAC	0.559	0.006
s_Parvimonas_micra	BAC	0.623	0.001
s_Olsenella_sp_oral_taxon_807	BAC	0.657	0.001
s_Streptococcus_cristatus	BAC	0.624	0.001
s_Blautia_sp_Marseille_P3087	BAC	0.568	0.005

Abbreviations: BAC, breath alcohol concentration at baseline (timepoint A); OCDS, obsessive compulsive drinking scale; PACS, penn alcohol craving scale.

### Correlation of different levels during the treatment

**FIGURE 4:**
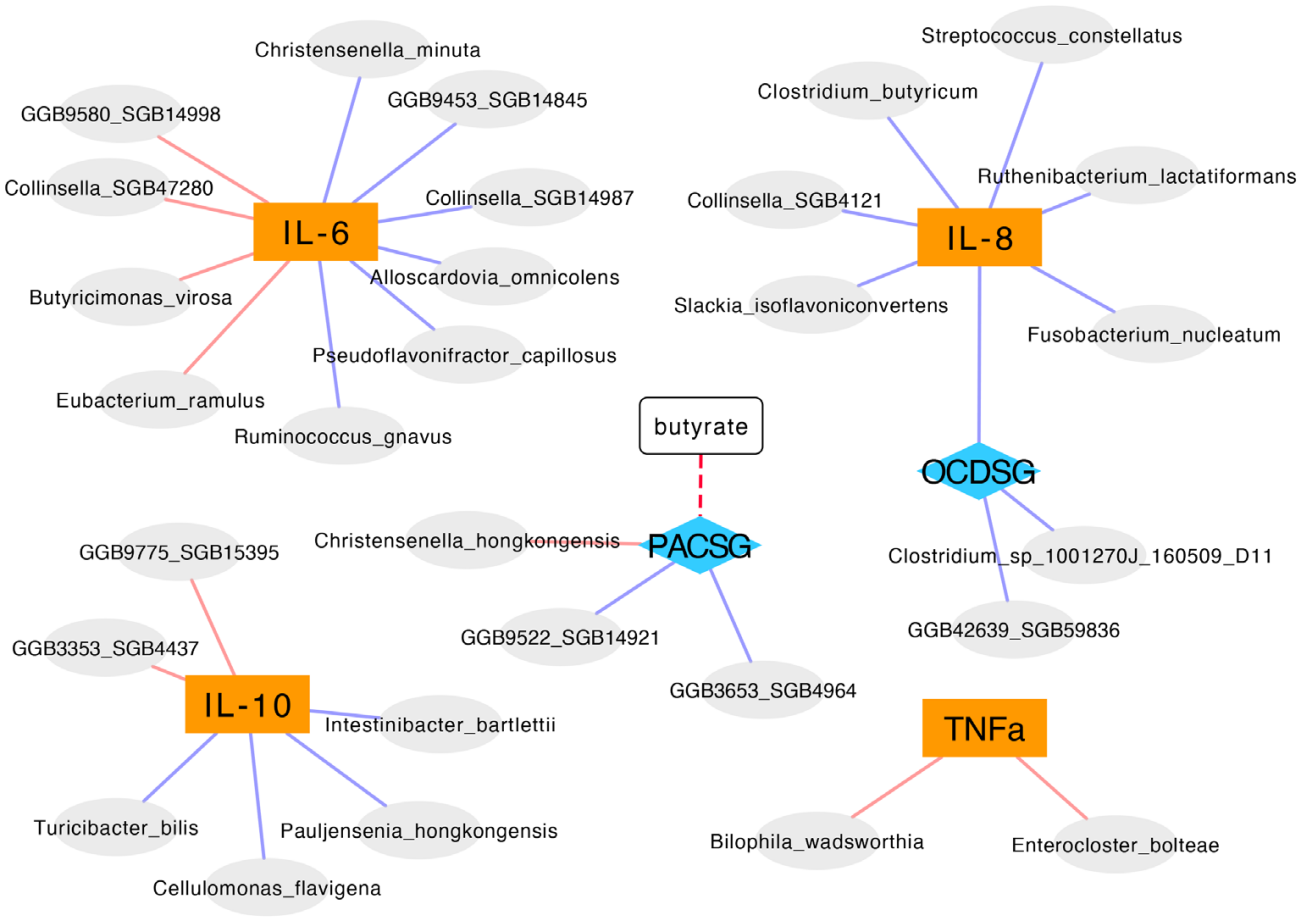
Correlation between changes of IL‐6, IL‐8, IL‐10 and TNF‐alpha, butyrate levels and craving scores (penn alcohol craving scale total score, PACSG, and obsessive compulsive drinking scale total score, OCDSG) during withdrawal therapy. Associations between changes (baseline vs. end of withdrawal therapy) of parameters of all levels investigated are shown. Colors of edges refer to positive (blue) and negative (red) correlations. The negative association between IL‐6 and the butyrate production potential only trended (*p* < 0.1) significant.

Changes in interleukin levels during withdrawal correlated with changes in the abundance of several bacterial species (Figure 4; Table 5). Furthermore, OCDS levels correlated negatively with IL‐8 levels and two species. PACS levels correlated with the abundances of three species. A decrease in PACS score trended to be associated with an increase in the butyrate production potential (*p* < 0.1).

**TABLE 5:** Correlation of changes from timepoint A to B.

Row	Column	*ρ*	*p*
IL‐8	OCDS	−0.565	0.003
s_GGB42639_SGB59836	OCDS	−0.477	0.006
s_Clostridium_sp_1001270J_160509_D11	OCDS	−0.452	0.009
s_Collinsella_SGB47280	IL‐6	−0.416	0.003
s_GGB9775_SGB15395	IL‐10	−0.413	0.004
s_Bilophila_wadsworthia	TNF‐α	−0.399	0.005
s_GGB9522_SGB14921	PACS	−0.398	0.008
s_Butyricimonas_virosa	IL‐6	−0.398	0.005
s_GGB3653_SGB4964	PACS	−0.394	0.008
s_Enterocloster_bolteae	TNF‐α	−0.392	0.006
s_Eubacterium_ramulus	IL‐6	−0.380	0.008
s_GGB3353_SGB4437	IL‐10	−0.380	0.008
s_GGB9580_SGB14998	IL‐6	−0.374	0.009
s_Collinsella_SGB14987	IL‐6	0.375	0.009
s_GGB9453_SGB14845	IL‐6	0.382	0.007
s_Fusobacterium_nucleatum	IL‐8	0.385	0.007
s_Slackia_isoflavoniconvertens	IL‐8	0.391	0.006
s_Clostridium_butyricum	IL‐8	0.391	0.006
s_Ruminococcus_gnavus	IL‐6	0.394	0.006
s_Pseudoflavonifractor_capillosus	IL‐6	0.395	0.006
s_Christensenella_minuta	IL‐6	0.411	0.004
s_Turicibacter_bilis	IL‐10	0.416	0.003
s_Cellulomonas_flavigena	IL‐10	0.424	0.003
s_Alloscardovia_omnicolens	IL‐6	0.427	0.002
s_Intestinibacter_bartlettii	IL‐10	0.433	0.002
s_Christensenella_hongkongensis	PACS	0.442	0.003
s_Pauljensenia_hongkongensis	IL‐10	0.444	0.002
s_Collinsella_SGB4121	IL‐8	0.468	0.001
s_Streptococcus_constellatus	IL‐8	0.474	0.001
s_Ruthenibacterium_lactatiformans	IL‐8	0.485	0.000

Abbreviations: IL, interleukin; OCDS, obsessive compulsive drinking scale; PACS, penn alcohol craving scale; s, species; TNF, tumor necrosis factor.

## DISCUSSION

Throughout alcohol withdrawal therapy, gut microbiota composition and the butyrate pathway changed and were linked with interleukin levels as well as behavioral parameters such as alcohol craving and alcohol intake. When compared to a healthy control group, which had significantly higher butyrate production capacity and species diversity, the community distance between timepoint A and B decreased significantly, demonstrating a change toward a healthier microbiota composition due to alcohol withdrawal.

As hypothesized and expected, the butyrate production capacity increased significantly throughout withdrawal, and microbiome diversity at the beginning of withdrawal correlated strongly with the butyrate pathway. As other research has shown that the abundance of butyrate‐producing bacteria directly leads to an increase in butyrate production (Kircher et al., 2022), it is fair to assume that this increase in production capacity directly translates into higher butyrate levels. In line with those results the abundance of *Eubacterium rectale* and *Faecalibacterium prausnitzii* increased throughout withdrawal, bacteria known for their ability to produce butyrate and to have positive health effects (Ferreira‐Halder et al., 2017; Karim et al., 2024) and anti‐inflammatory properties (Lu et al., 2022; Sokol et al., 2008). *F. prausnitzii* was already described as being decreased in AUD and was associated with high intestinal permeability (Leclercq, Matamoros, et al., 2014). In the “Lunar Palace 365” study, a 1‐year‐long isolation study that took place in Luna Palace 1, both bacteria were among the four that were regarded as “potential psychobiotics” due to their association with psychological changes in the long‐term closed environment (Hao et al., 2023). In one study by Leclercq et al. (Leclercq, Matamoros, et al., 2014), *F. prausnitzii* was negatively associated with IL‐8 levels, which was not the case in the present study. However, in contrast to the study by Leclercq, Matamoros, et al., 2014, which used 16S rRNA gene sequencing for determining gut microbiota composition and where *F. prausnitzii* levels remained unchanged at the end of detoxification, we found increased levels after withdrawal therapy. Our study, therefore, adds new evidence to *E. rectales'* and *F. prasunitzii's* role in mental disorders. Furthermore, we found a negative association between changes in craving (as assessed with the PACS) and changes in butyrate. One core physiological function linking butyrate and alcohol craving might be appetite regulation. Studies have shown that butyrate influences food intake by decreasing appetite and affects central appetite regulation before entering the bloodstream (Fluitman et al., 2018; Li et al., 2018). Leptin and ghrelin, critical players in appetite regulation, have an influence on drug reward (Dickson et al., 2011; DiLeone, 2009; Shen et al., 2016) and their function is linked to drug craving (Bach et al., 2021; Koopmann et al., 2018). In one study in AUD patients, leptin and acylated ghrelin were shown to have opposing effects on mesolimbic cue‐reactivity and alcohol craving (Bach et al., 2019). Ghrelin was shown to modulate mesolimbic reactivity to alcohol cues (Koopmann et al., 2019), while in one randomized controlled trial, leptin levels were reduced by intravenous ghrelin administration and correlated with cue‐induced alcohol craving (Haass‐Koffler et al., 2015). Butyrate regulates leptin expression through adipocytes (Soliman et al., 2011) and reduces fasting insulin and leptin levels when SCFAs are supplemented (Lin et al., 2012). Only a few studies have yet investigated the association of butyrate with alcohol craving. For example, one study in young binge drinkers reported that reduced butyrate synthesis was associated with higher craving levels (Carbia et al., 2023). Furthermore, one metagenomic study reported that AUD was inversely associated with the levels of butyrate‐producing species from the *Clostridiales* order (Dubinkina et al., 2017). Its potential use as a therapeutic tool has been shown in a randomized controlled trial in AUD patients with concomitant cirrhosis who were treated with fecal microbiota. While having several effects, fecal microbiota treatment reduced craving, increased microbial diversity, and SCFA‐producing taxa (Bajaj et al., 2021). In summary, our study adds evidence to the assumption that butyrate affects alcohol craving, possibly via its effect on appetite regulation.

The abundance of *R. gnavus* decreased significantly during alcohol withdrawal therapy. *R. gnavus* is known for mucus degradation and a negative effect on epithelial integrity (Crost et al., 2023). In one recent study on the effect of alcohol on gut microbiota, the abundance of *R. gnavus* was positively associated with alanine aminotransferase (ALT) and aspartate aminotransferase (APT) suggesting an association with liver inflammation (Jiao et al., 2022). With an increasing number of studies being published on *R. gnavus*, one recent and thorough review highlighted its association with metabolic diseases such as obesity, diabetes mellitus type 2, and nonalcoholic fatty liver disease (NAFLD), pointing to its role in the gut–liver–brain axis (Crost et al., 2023). While it is often referred to as a pro‐inflammatory microbe, the authors argue that these effects are often strain‐specific and context‐dependent and, therefore, do not generally regard *R. gnavus* as a microbe negatively associated with intestinal function and human health (Crost et al., 2023). Since the abundance of *R. gnavus* decreased while butyrate function increased, our results suggest an association of *R. gnavus* with alcohol use. Our study, therefore, adds to the evidence that *R. gnavus* seems to be a microbe influencing health and disease (Juge, 2023).

One aim of the present study was to replicate some of the previous findings by Leclercq, De Saeger, et al. (2014), where they found IL‐8 levels to be positively correlated with alcohol craving. In the present study, IL‐8 changes throughout withdrawal positively correlated with craving changes (OCDS) throughout withdrawal, adding evidence to IL‐8's potential role in neuroinflammation associated with alcohol craving. In the context of alcohol consumption, increased IL‐8 levels were identified in persons drinking higher amounts of alcohol, especially in those with AUD (Gonzalez‐Quintela et al., 2007). Several studies have described a link between interleukin levels and alcohol use (Gonzalez‐Quintela et al., 2007; González‐Quintela et al., 2000; Hillmer et al., 2020). In our study, however, we integrated interleukin levels into a model of the microbiome‐gut‐brain axis and showed an association between them and specific microbiome composition, as well as alcohol craving. Thus, we gather new evidence on possible causal relationships between the gut microbiome and clinical parameters, possibly via inflammation.

Naturally, the study has several limitations. Since we focused on functional and diversity changes throughout alcohol withdrawal and their association with interleukins and craving, we did not include a healthy control group directly matched to our sample. Furthermore, while the control group was free of liver disease (Kircher et al., 2022), we have information neither on existing mental disorders nor on interleukin levels. While it is useful to demonstrate a shift of microbiota composition toward a healthier state, comparisons with a larger and well‐matched healthy control group will probably provide more detailed insights into species and functions that are altered in AUD. Another limitation is the time interval studied. In Germany, qualified withdrawal therapy usually lasts 14 to 21 days, while the latter applies typically to patients having their first qualified withdrawal therapy. Most patients in our sample had multiple withdrawal attempts, and thus, the therapy lasted shorter. Hence, some nonobserved changes could only be aberrant since the time interval was too short, and the changes found need to be interpreted with caution. Furthermore, while we excluded patients with liver cirrhosis and acute steatohepatitis through the assessment of patient history, clinical investigation, and interview, we did not conduct apparative diagnostics to assess liver steatosis and cirrhosis (e.g., fibroscan). Another limitation is that our results are correlative in nature; therefore, definitive causal statements about a possible effect of microbiome function and diversity on alcohol craving cannot be drawn. Another limitation is missing measurements of gut barrier integrity and intestinal permeability, as others have reported a so‐called leaky gut for AUD and an amelioration during alcohol withdrawal (Leclercq et al., 2012; Leclercq, Matamoros, et al., 2014).

In summary, alcohol withdrawal therapy had a direct effect on gut microbiota composition and butyrate production and promotes a shift toward a healthier microbiome. Some bacterial taxa were associated with interleukin levels as well as behavioral parameters such as alcohol craving and alcohol intake. We discuss why the increase in butyrate production could be a factor linking the behavioral and physiological characteristics of AUD with neuroinflammation. While we did not find a link between butyrate and alcohol craving at baseline, changes in IL‐8 were linked to alcohol craving. Furthermore, we found a trend of negative association between craving and butyrate production. Our study, therefore, adds new evidence that helps us understand the microbiome–gut–brain axis.

## CONFLICT OF INTEREST STATEMENT

The authors have no conflicts of interest.

## Supporting information


Figure S1.

## Data Availability

The data that support the findings of this study are available on request from the corresponding author. The data are not publicly available due to privacy or ethical restrictions.
