# Supplementary figures and images for: The effect of alcohol withdrawal therapy on gut microbiota in alcohol use disorder and its link to inflammation and craving
Source: Alcohol Clin Exp Res (Hoboken). 2025 Aug 23;49(9):1912–23. doi: 10.1111/acer.70128 (PMC12463751; doi:10.1111/acer.70128)

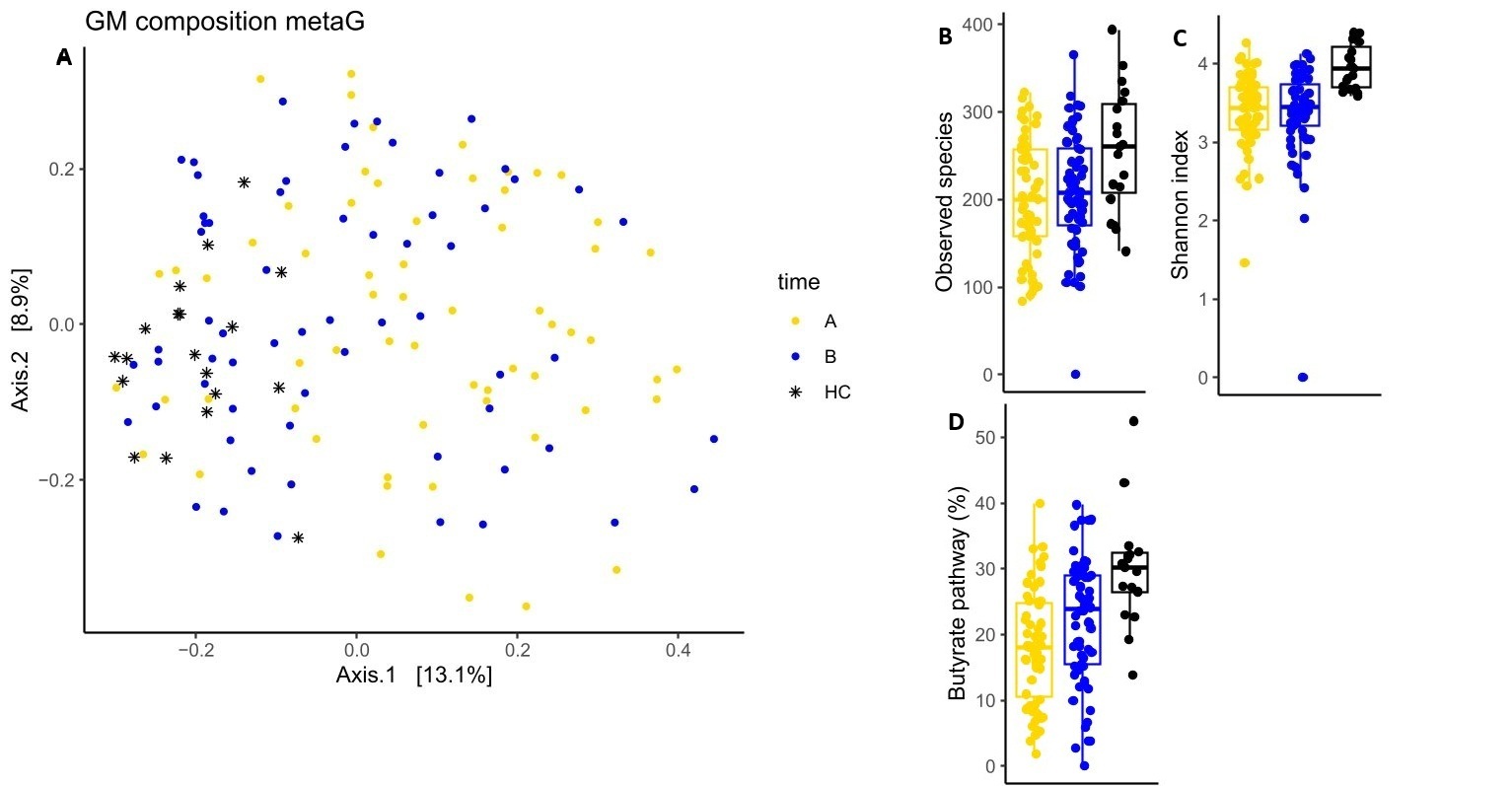

Supplement: Supplementary file 1 — Figure S1. [file ACER-49-1912-s001.jpg]
